# The Effect of a Tobacco Use Reduction Program on the Prevalence of Smoking and Tobacco Use and Quitting Behavior Among People Living With HIV/AIDS in Michigan

**DOI:** 10.5888/pcd21.230115

**Published:** 2024-01-11

**Authors:** Farid Shamo, Kathryn E. Macomber, Julia Hitchingham, Sean Bennett, Sheyonna Watson

**Affiliations:** 1 Michigan Department of Health and Human Services, Lansing, Michigan

## Abstract

HIV has evolved from a serious infectious disease to a manageable chronic disease. Tobacco use has a devastating effect on the health of people living with HIV/AIDS (PLWH). The Michigan Tobacco Use Reduction Program for PLWH was established in 2015 to learn about tobacco use among PLWH, gather information on entities that provide health care services to PLWH, and improve tobacco treatment services for this population. The program offers evidence-based treatment interventions to all PLWH who are tobacco users, eligible for the Ryan White HIV/AIDS Program, and served by AIDS service organizations in Michigan. This evaluation had 3 primary outcomes: 1) rates of smoking and tobacco use among program clients, 2) the percentage of clients who made a quit attempt in the previous 12 months, and 3) the types of tobacco cessation methods used by clients. All data were self-reported in 3 surveys, one each in 2015, 2017, and 2021. The rate of cigarette smoking overall among clients decreased significantly from 49.5% in 2015 to 41.5% in 2017. The percentage of clients who made a quit attempt increased from 37.0% in 2015 to 41.9% in 2017; in 2021, this rate was 54.4%. By age, in all 3 survey years, the highest rate of tobacco use was among clients aged 35 to 44 years (range, 48.4%–57.4%). Smoking rates declined significantly from 2015 to 2017 among African American (50.5% to 42.8%) and White clients (49.8% to 39.9%). The most frequently used method of tobacco cessation was medications prescribed by a physician (range, 20%–30%). State tobacco control programs are encouraged to collaborate with their state HIV/AIDS bureaus to create similar programs to treat tobacco use among PLWH.

**Summary**

**What is already known on this topic?**

Smoking prevalence among people living with HIV/AIDS (PLWH) is estimated to be 2 to 3 times higher than in the general population, and those who smoke in this population lose an average 12.3 life-years more than those who do not smoke.

**What is added by this report?**

Tobacco use reduction programs embedded in AIDS service organizations can increase the rate of quit attempts and significantly reduce the rate of tobacco use among PLWH through evidence-based treatment interventions.

**What are the implications for public health practice?**

US tobacco control programs are encouraged to collaborate with their state HIV/AIDS bureaus to create similar programs to offer tobacco use reduction services to PLWH.

## Introduction

Smoking is the leading cause of preventable death and disease in the US. Cigarette smoking is responsible for more than 480,000 US deaths annually, including more than 41,000 deaths resulting from secondhand smoke exposure, which translates to approximately 1 in 5 deaths annually, or 1,300 deaths every day (1). In Michigan each year, 16,200 adults die as a result of smoking (1). The prevalence of smoking among adults in Michigan in 2021 was 17.0%, higher than the national average of 14.4% (2). Furthermore, the smoking prevalence among people living with HIV/AIDS (PLWH) is estimated to be more than 2 to 3 times higher than the rate among the general population (3).

Since the introduction of antiretroviral therapy, HIV-associated illness and death rates have declined and the life expectancy of PLWH with good adherence to medical guidance and care has increased. HIV is no longer considered a terminal disease but is now a manageable chronic disease. Lifestyle factors, such as lack of exercise, poor diet, and tobacco use are among the most prevalent problems among PLWH. Smoking and tobacco use have had a devastating effect on the health of this population. Because of the effects of HIV on a person’s immune system and inflammatory processes, smoking can result in a higher risk for cancer, chronic obstructive pulmonary disease, heart disease, stroke, HIV-related infections, and bacterial pneumonia (4,5). Among HIV patients receiving well-organized care with free access to antiretroviral therapy, those who smoke lose about 12.3 more years of life than those who never smoked (6,7). Treating PLWH for tobacco dependence is more difficult than treating members of the general population because of the many stresses facing PLWH, including stigma and prejudice. However, some evidence indicates that most PLWH try to improve their health after a diagnosis of HIV (8).

In Michigan, according to the Medical Monitoring Project for 2014, the smoking rate among PLWH was estimated to be 52% (9). This rate was the highest reported for any underrepresented population in Michigan; for example, the smoking rate among the African American population in Michigan was 26.6%, and among the lesbian/gay/bisexual (LGB) population in Michigan it was 36.7% (9). To address these health disparities, the Michigan Tobacco Control Program formed a partnership with the Ryan White HIV/AIDS Program in Michigan in January 2015, the first of its kind nationally. Initially, the Michigan Tobacco Control Program contracted with 16 AIDS service organizations (ASOs) in 2015 to provide evidence-based treatment interventions, such as nicotine replacement therapy, motivational interviewing, and cognitive behavioral therapy; this number expanded to 19 contracts in 2022.

## Purpose and Objectives

The objectives of the Michigan Tobacco Use Reduction Program for PLWH are to learn more about PLWH and their attitudes and behaviors toward tobacco use, gather information on the entities that provide health care services to this population, and improve tobacco treatment services for this population. The program uses evidence-based tobacco treatment best practices and promotes changes in health systems in the following areas: tobacco screening, referral, and cessation services; treatment planning; and follow-up.

## Intervention Approach

The Michigan Tobacco Use Reduction Program for PLWH offers evidence-based treatment interventions, such as nicotine replacement therapy, motivational interviewing, and cognitive behavioral therapy, to all PLWH who are eligible for the Ryan White HIV/AIDS Program and served by ASOs in Michigan. These interventions consist of individual counseling (with uptake among clients ranging from 41% to 48% over time), group counseling (with uptake among clients ranging from 10.6% to 11.4% over time), health education classes (with uptake among clients ranging from 14% to 18% over time), and precontemplation workshops (uptake not measured).

Clients of the Michigan Tobacco Use Reduction Program for PLWH have unlimited access to tobacco treatment specialists for ongoing counseling and support. Treatment plans are created collaboratively between tobacco treatment specialists and clients. Tobacco treatment specialists receive training in culturally appropriate tobacco dependence treatment, trauma-informed care, health equity and social justice, addressing implicit bias, and racial equity. Tobacco treatment specialists use the Fagerström Test for Nicotine Dependence (10) to assess smokers for nicotine dependency and the DSM-5 screening to assess nicotine withdrawal (11) and provide referrals for ongoing counseling and behavioral health interventions. The program offers all 7 tobacco dependence treatment medications (eg, varenicline, bupropion) approved by the US Food and Drug Administration. The cost of these medications is covered through the Michigan Drug Assistance Program.

## Evaluation Approach

This evaluation of the effect of the Michigan Tobacco Use Reduction Program for PLWH had 3 primary outcomes: 1) the rates of smoking and tobacco use among clients, 2) the percentage of clients who made a quit attempt in the previous 12 months, and 3) the types of tobacco cessation methods used (types of counseling enrolled in and medications used [both physician-prescribed and over-the-counter]). All data were self-reported in 3 surveys.

### Surveys on tobacco use–related knowledge, attitudes, and behaviors

To establish baseline data before implementing the intervention, we surveyed clients from March through June 2015 to assess their tobacco use–related knowledge, attitudes, and behaviors. The 27-item survey was reviewed and approved by the institutional review board of the Michigan Department of Health and Human Services. Inclusion criteria for survey participation was being a PLWH at a partnering ASO and being aged 18 years or older. We conducted a second survey (in 2017) that used similarly worded questions and response options; these surveys assessed smoking rates. A third survey (in 2021) differed slightly from the first 2 surveys; instead of smoking rates, for example, this survey assessed tobacco use rates. We collected data on sex and gender, age, sexual orientation, and race and ethnicity.

All survey participants were recruited by the ASOs. In the first survey, 1,478 of 2,997 clients participated. In the second survey, 1,485 of 3,857 clients participated, and in the third survey, 902 of 7,100 clients participated. To put these sample sizes in context, Michigan had 15,629 cases of HIV infection as of January 1, 2017 (12). The demographic characteristics of the participants were similar in the 3 surveys (Table 1).

**Table 1:** Demographic Characteristics of Survey Participants in 3 Survey Years, Michigan Tobacco Use Reduction Program for PLWH

Characteristic	No./total (%)^a^			PLWH in Michigan in 2017, % (N = 15,629)^b^
	2015 Survey (N = 1,478)	2017 Survey (N = 1,485)	2021 Survey (N = 902)	
**Sex and gender**				
Male	1,012/1,380 (73.3)	1,082/1,415 (76.5)	600/881 (68.1)	78
Female	350/1,380 (25.4)	312/1,415 (22.0)	252/881 (28.6)	21
Transgender	18/1,380 (1.3)	21/1,415 (1.5)	29/881 (3.3)	1
**Age group, y**				
18–24	152/1,396 (10.9)	128/1,471 (8.7)	25/877 (2.9)	NA
25–34	268/1,396 (19.2)	271/1,471 (18.4)	149/877 (17.0)	NA
35–44	282/1,396 (20.2)	283/1,471 (19.2)	161/877 (18.4)	NA
≥45	694/1,396 (49.7)	789/1,471 (53.6)	542/877 (61.8)	NA
**Sexual orientation**				
Heterosexual	581/1,388 (41.9)	554/1,438 (38.5)	390/857 (45.5)	NA
LGB	807/1,388 (58.1)	884/1,438 (61.5)	467/857 (54.5)	NA
**LGB group**				
Lesbian	14/1,337 (1.0)	10/1,344 (0.7)	3/881 (0.3)	NA
Gay	642/1,337 (48.0)	674/1,344 (50.1)	388/881 (44.0)	NA
Bisexual	103/1,337 (7.7)	121/1,344 (9.0)	76/881 (8.6)	NA
**Race and ethnicity^c^**				
African American	640/1,422 (45.0)	710/1,479 (48.0)	419/855 (49.0)	56
Asian	19/1,422 (1.3)	20/1,479 (1.4)	11/855 (1.3)	1
Hispanic	71/1,422 (5.0)	67/1,479 (4.5)	77/855 (9.0)	5
Native American	56/1,422 (3.9)	44/1,479 (3.0)	14/855 (1.6)	1
White	741/1,422 (52.1)	649/1,479 (43.9)	359/855 (42.0)	34

Abbreviations: LGB, lesbian, gay, bisexual; NA, not available; PLWH, people living with HIV/AIDS.

a Not all survey participants answered all survey questions. Percentages were calculated according to the number of participants who answered question. Percentages may not add to 100 because of rounding.

b Data source: Michigan Department of Health & Human Services (12).

c Some participants selected >1 race and ethnicity.

**Smoking and tobacco use.** The survey asked respondents about their smoking status. The response options were current smoker, former smoker, and never smoked. Smoking was defined as including cigarettes, cigars, cigarillos, and pipes.

**Quit attempts.** The survey question on quit attempts was, “During the past 12 months, have you stopped smoking/tobacco use for one day or longer because you were trying to quit?” Respondents answered yes or no.

**Types of cessation methods used.** The question was, “Have you used or enrolled in any of the following quit smoking/tobacco methods?” Respondents were asked to mark all that apply: any type of counseling, medications prescribed by a physician, over-the-counter medications, telephone quitline, stop-smoking classes, quit on your own (“cold turkey”), and none.

### Analysis

In this evaluation, we report only the results of these 3 outcomes in the 3 survey years: rates of smoking and tobacco use, quit attempts, and types of tobacco cessation methods used. We did not link data on individual clients from survey to survey, nor did we follow up with each survey participant to complete all 3 surveys. However, the same ASOs with their clients participated in the 3 surveys, so many clients could have participated in 2 or even 3 surveys. We compared survey results for 2015, 2017, and 2021. We stratified data by age group, sex and gender, sexual orientation, and race and ethnicity. We used χ^2^ tests to determine significant differences between 2015 and 2017; *P* < .05 was considered significant. We did not conduct tests of differences between the 2021 survey and the 2015 or 2017 survey because of changes in survey questions and response options. We used SPSS version 28 (IBM Corporation) to conduct all analyses.

## Results

Our main finding was the significant reduction in the rate of cigarette smoking from 49.5% in 2015 to 41.5% in 2017 (*P* < .001) (Table 2). We also found a significant increase in the percentage of clients who made a quit attempt, from 37.0% in 2015 to 41.9% in 2017; in 2021, this rate was 54.4%.

**Table 2:** Rates of Smoking and Tobacco Use, by Demographic Characteristics, Michigan Tobacco Use Reduction Program for People Living With HIV/AIDS

Characteristic	No./total (%)		
	2015 Survey on smoking	2017 Survey on smoking	2021 Survey on tobacco use^a^
**Overall**	701/1,417 (49.5)	608/1,467 (41.5)^b^	363/876 (41.4)
**Sex and gender**			
Male	513/1,012 (50.7)	427/1,082 (39.5)^b^	240/600 (40.0)
Female	161/350 (46.0)	123/312 (39.4)	108/252 (42.9)
Transgender	12/18 (66.7)	14/21 (66.7)	20/29 (69.0)
**Age group, y**			
18–24	74/152 (48.7)	42/128 (32.8)^b^	8/25 (32.0)
25–34	139/268 (51.9)	122/271 (45.0)	70/149 (47.0)
35–44	162/282 (57.4)	137/283 (48.4)^b^	82/161 (50.9)
≥45	322/694 (46.4)	296/789 (37.5)^b^	206/542 (38.0)
**Sexual orientation**			
Heterosexual	293/581 (50.4)	241/554 (43.5)^b^	176/390 (45.1)
Lesbian, gay, or bisexual	398/807 (49.3)	346/884 (39.1)^b^	182/467 (39.0)
**Race and ethnicity^c^**			
African American	323/640 (50.5)	304/710 (42.8)^b^	182/419 (43.4)
Hispanic	31/71 (43.7)	30/67 (44.8)	29/77 (37.7)
Native American	32/56 (57.1)	22/44 (50.0)	7/14 (50.0)
White	369/741 (49.8)	259/649 (39.9)^b^	155/359 (43.2)
**Quit attempts**			
Quit attempts in the past 12 months	272/735 (37.0)	269/642 (41.9)^b^	200/368 (54.4)

Abbreviation: LGB, lesbian, gay, bisexual.

a Tests of differences between 2021 survey and the 2015 and 2017 surveys were not conducted because of changes in survey questions and response options.

b Differences between 2015 and 2017 are significant at *P* < .05; determined by χ^2^ test.

c Because the number of Asians who reported smoking was <5, we excluded this group from this analysis.

By age, in all 3 survey years, the highest rate of tobacco use was among clients aged 35 to 44 years (range, 48.4%–57.4%). We found a significant decrease in smoking rates from 2015 to 2017 in all age groups except the group aged 25 to 34 years. In this age group, the smoking rate was 51.9% in 2015 and 45.0% in 2017, and the tobacco use rate was 47.0% in 2021. We also found significant reductions in smoking rates in the 2 sexual orientation groups: in the heterosexual group, rates decreased from 50.4% in 2015 to 43.5% in 2017, and in the LGB group from 49.3% to 39.1% (*P* < .001 for both). In 2021, among clients who identified as heterosexual, 45.1% were tobacco users, as were 39.0% of LGB clients.

Among men, smoking rates decreased significantly from 50.7% in 2015 to 39.5% in 2017. In 2021, 40.0% of men reported tobacco use. Differences in smoking rates between 2015 and 2017 were not significant in the female and transgender groups. In 2021, 42.9% of women and 69.0% of transgender clients were tobacco users.

Smoking rates declined significantly from 2015 to 2017 among African American (50.5% to 42.8%) and White clients (49.8% to 39.9%) but not among Hispanic (43.7% to 44.8%) or Native American (57.1% to 50.0%) clients. In 2021, the prevalence of tobacco use was 43.4% among African American, 43.2% among White clients, 37.7% among Hispanic clients, and 50.0% among Native American clients (Table 2).

We found that the most frequently used method of cessation was medications prescribed by a physician. We also found an upward trend in the use of any type of counseling: 2.8% in 2015, 14.9% in 2017, and 20.9% in 2021. The percentage of clients who used medications prescribed by physicians was significantly higher in 2017 (27.9%) than in 2015 (20.1%). The use of tobacco cessation classes also increased: 3.6% of clients indicated enrolling 2015, 8.9% in 2017, and 11.6% in 2021. We did not observe major changes in use of the state quit line, over-the-counter medications, or quitting on one’s own (“cold turkey”). These rates, respectively, were 40.5% in 2015, 33.8% in 2017, and 31.9% in 2021.

## Implications for Public Health

To our knowledge, this is the first study to assess smoking and tobacco use and quitting behavior among PLWH after implementation of evidence-based treatment interventions. We found no published studies of long-term, permanent, ongoing cessation programs for treating PLWH who use tobacco. Several studies addressed smoking cessation among PLWH; although the researchers used cessation methods that were similar to those used in our program, their interventions had a shorter time frame and had a smaller number of patients (13–15).

This evaluation has several potential limitations. We used point-in-time surveys, which could underrepresent or overrepresent the overall effect of our program. The surveys were not longitudinal, so clients participating in the first survey did not necessarily participate in the second or third surveys. In addition, selection bias may have occurred because participants were not randomly selected. Finally, the survey in 2021 was conducted during the COVID-19 pandemic, and we do not know how the pandemic moderated services, stressors, or nicotine dependence habits.

The prevalence of smoking and tobacco use is higher among PLWH than among the general population, which necessitates providing evidence-based tobacco treatment services to this population. Creating a tobacco use reduction program within ASOs can significantly reduce the rates of smoking and tobacco use among PLWH. State tobacco control programs are encouraged to collaborate with their state HIV/AIDS bureaus to create similar programs to treat tobacco use among PLWH.

## References

[1] US Department of Health and Human Services. *The Health Consequences of Smoking — 50 Years of Progress: A Report of the Surgeon General.* US Department of Health and Human Services, Centers for Disease Control and Prevention, National Center for Chronic Disease Prevention and Health Promotion, Office on Smoking and Health; 2014.

[2] Centers for Disease Control and Prevention. BRFSS prevalence & trends data. Last reviewed July 19, 2023. Accessed August 2, 2023. https://nccd.cdc.gov/BRFSSPrevalence

[3] Mdodo R , Frazier EL , Dube SR , Mattson CL , Sutton MY , Brooks JT , . Cigarette smoking prevalence among adults with HIV compared with the general adult population in the United States: cross-sectional surveys. *Ann Intern Med.* 2015;162(5):335–344. 10.7326/M14-0954 25732274

[4] Samji H , Cescon A , Hogg RS , Modur SP , Althoff KN , Buchacz K , ; North American AIDS Cohort Collaboration on Research and Design (NA-ACCORD) of IeDEA. Closing the gap: increases in life expectancy among treated HIV-positive individuals in the United States and Canada. *PLoS One.* 2013;8(12):e81355. 10.1371/journal.pone.0081355 24367482 PMC3867319

[5] Lifson AR , Neuhaus J , Arribas JR , van den Berg-Wolf M , Labriola AM , Read TR ; INSIGHT SMART Study Group. Smoking-related health risks among persons with HIV in the Strategies for Management of Antiretroviral Therapy clinical trial. *Am J Public Health.* 2010;100(10):1896–1903. 10.2105/AJPH.2009.188664 20724677 PMC2936972

[6] Helleberg M , Afzal S , Kronborg G , Larsen CS , Pedersen G , Pedersen C , . Mortality attributable to smoking among HIV-1-infected individuals: a nationwide, population-based cohort study. *Clin Infect Dis.* 2013;56(5):727–734. 10.1093/cid/cis933 23254417

[7] Deeks SG , Tracy R , Douek DC . Systemic effects of inflammation on health during chronic HIV infection. *Immunity.* 2013;39(4):633–645. 10.1016/j.immuni.2013.10.001 24138880 PMC4012895

[8] Ng YC , Caires A . The health promotion model in HIV care. *Aquichan.* 2016;16(4):418–429. 10.5294/aqui.2016.16.4.2

[9] Michigan.gov. *MMP Surveillance Summary Report, 2014: Behavioral and Clinical Characteristics of Persons Receiving Medical Care for HIV Infection — Comparison of Michigan and National Medical Monitoring Project Data for 2014.* Accessed October 25, 2023. https://www.michigan.gov/-/media/Project/Websites/mdhhs/Folder3/Folder71/Folder2/Folder171/Folder1/Folder271/MMP_SS_Report.pdf

[10] National Institutes of Health, National Institute on Drug Abuse. NIDA CTN common data elements. Instrument: Fagerstrom Test For Nicotine Dependence (FTND). Accessed October 25, 2023. https://cde.nida.nih.gov/instrument/d7c0b0f5-b865-e4de-e040-bb89ad43202b

[11] Halberstadt AL , Skrzynski CJ , Wright AGC , Creswell KG . Predicting smoking and nicotine dependence from the DSM-5 alternative model for personality pathology. *Pers Disord.* 2022;13(1):84–95. 10.1037/per0000487 33705195 PMC8916785

[12] Michigan Department of Health & Human Services. *Michigan Statewide HIV Surveillance Report: New Diagnoses and Prevalence Tables.* July 1, 2017. Accessed July 20, 2022. https://www.michigan.gov/documents/mdhhs/Michigan_Statewide_HIV_Surveillance_Report_602596_7.pdf

[13] Cropsey KL , Hendricks PS , Jardin B , Clark CB , Katiyar N , Willig J , . A pilot study of screening, brief intervention, and referral for treatment (SBIRT) in non-treatment seeking smokers with HIV. *Addict Behav.* 2013;38(10):2541–2546. 10.1016/j.addbeh.2013.05.003 23787030 PMC3725186

[14] Matthews AK , Conrad M , Kuhns L , Vargas M , King AC . Project Exhale: preliminary evaluation of a tailored smoking cessation treatment for HIV-positive African American smokers. *AIDS Patient Care STDS.* 2013;27(1):22–32. 10.1089/apc.2012.0253 23305259

[15] Fuster M , Estrada V , Fernandez-Pinilla MC , Fuentes-Ferrer ME , Tellez MJ , Vergas J , . Smoking cessation in HIV patients: rate of success and associated factors. *HIV Med.* 2009;10(10):614–619. 10.1111/j.1468-1293.2009.00735.x 19659946

